# Effects of phosphorylation-modified long-chain inulin on wheat starch: Physicochemical properties and retrogradation behaviors

**DOI:** 10.1016/j.fochx.2024.101860

**Published:** 2024-09-30

**Authors:** Ruijie Zhang, Denglin Luo, Chonghui Yue, Zhouya Bai, Peiyan Li, Libo Wang, Sihai Han

**Affiliations:** a College of Food and Bioengineering, Henan University of Science & Technology, 471023 Luoyang, Henan Province, China; b Henan Engineering Research Center of Food Material, Henan University of Science & Technology, 471023 Luoyang, Henan Province, China

**Keywords:** Long-chain inulin, Phosphorylation modification, Wheat starch, Physicochemical properties, Retrogradation behaviors

## Abstract

In this research, modification of long-chain inulin (FXL) through phosphorylation (PFXL) to enhance its application in wheat starch (WS) and starch-based products. The impacts of PFXL on the pasting, rheology, microstructure, and retrogradation characteristics of WS were researched. The findings revealed that PFXL significantly reduced both the breakdown and setback values of WS. Additionally, the incorporation of PFXL reduced the viscoelasticity of WS paste and improved its fluidity. Scanning electron microscopy indicated that higher PFXL levels (>5 %) produced small fragments that partially covered the three-dimensional honeycomb structure of WS paste, thereby reducing water loss during short-term storage. PFXL also altered water distribution in WS gels, depending on concentration and storage duration. X-ray diffraction and Fourier-transform infrared spectroscopy suggested that PFXL effectively inhibited amylopectin recrystallization. Compared to FXL, PFXL exhibited a more pronounced ability to inhibit the aging of WS in short- and long-term storage.

## Highlights

• Phosphorylated long-chain inulin (PFXL) significantly inhibited the short-term retrogradation of wheat starch (WS).
• PFXL showed a better effect than long-chain inulin (FXL) in preventing the increase of gel hardness.
• During long-term aging, PFXL decreased the migration of free water in WS gel.
• Compared to FXL, PFXL prevented retrogradation of amylopectin during long-term storage.

## 1 Introduction

Wheat starch (WS), which constitutes approximately 85 % of the dry weight of wheat flour (Ma et al., 2021), plays a pivotal role in determining the quality of products made from WS. Nevertheless, WS presents certain inherent disadvantages in its application, including heat instability, poor solubility, and a tendency to retrograde, which restricts its suitability in foods (Chen, Fu & Luo, 2015; Zhao et al., 2017). In recent years, adding non-starch polysaccharides, such as inulin, laminaria japonica polysaccharide, and arabinoxylan, to enhance WS processing properties has attracted wide attention. Research has demonstrated that these non-starch polysaccharides reduced peak viscosity, breakdown, and setback values of WS while enhancing its gelling properties by lowering gel hardness and increasing water-holding capacity. Furthermore, they effectively inhibited WS retrogradation (Luo et al., 2017; Xie et al., 2022; Zhou et al., 2022).

Long-chain inulin (FXL) is a natural fructose polymer characterized by an average degree of polymerization (DP) exceeding 23 (Lou et al., 2022). Due to its higher average degree of polymerization compared to short-chain inulin (DP ≤ 10; FS) and natural inulin (DP 2–60; FI), FXL exhibits excellent gelling and emulsion stability. Starch gels containing FXL exhibited higher elastic (G′) and viscous moduli (G″) than those containing FS (Ji et al., 2020; Wang et al., 2019). Compared to FS and FI, FXL was also found to more effectively reduce the breakdown value (BD) of WS during gelatinization and improved the thermal stability of WS paste (Luo et al., 2017; Witczak et al., 2014). However, due to its longer molecular chains and fewer exposed hydroxyl groups, FXL had lower hydrophilicity, restricting its capability to prevent WS retrogradation and constraining its application in WS-based food production (Kou et al., 2019; Luo et al., 2017; Ye et al., 2018). FXL was found to significantly impede dough fermentation and elevate the firmness of steamed bread even at low addition levels (≤2.5 %) (Kou et al., 2019).

Due to the above limitations of FXL, it is necessary to modify it to expand its application in foods. Phosphorylation is considered a safe and common modification method for polysaccharides, in which phosphate groups react with hydroxyl groups of polysaccharides to form derivatives (Liu et al., 2022). This modification improved solubility, water retention, and resistance to the retrogradation of starches, including chestnut, maize, and rice starches (Jang et al., 2020; Liu et al., 2022). Our previous research demonstrated that phosphorylated FXL (PFXL) exhibited a more uniform particle size distribution and lower relative crystallinity (Tang et al., 2024). Moreover, PFXL displayed both hydrophilic and hydrophobic properties, facilitating the cross-linking of the gluten network and the formation of disulfide bonds (-SS). Therefore, it enhanced the gas retention and specific volume of steamed bread (Zhao et al., 2022; Zhao et al., 2023).

Generally, the physicochemical properties of PFXL and its use in flour-based products have been investigated (Tang et al., 2024; Zhao et al., 2022; Zhao et al., 2023). Nevertheless, the impact of PFXL on WS characteristics, particularly in terms of retrogradation, remains unexplored. Therefore, this research investigated the changes in WS properties before and after incorporating PFXL to understand the interaction of PFXL and WS. These findings provided valuable insights for extending the application of FXL in WS-based foods.

## 2 Materials and methods

### 2.1 Materials

Wheat flour (protein, fat, ash, and moisture content of 12.48 %, 0.51 %, 0.56 %, and 11.82 %, respectively) was sourced from Lvyuan stone mill grain processing company (Luoyang, China). Long-chain inulin (FXL, average DP ≥ 23, purity≥99 %) was purchased from Cosucra (Belgium). WS was extracted from wheat flour using the method of Martin with slight modifications (WS content of 87.9 %).

### 2.2 Preparation of PFXL

PFXL was synthesized following the process by Tang et al. (2024) with slight modifications. Disodium hydrogen phosphate (0.5 g), sodium dihydrogen phosphate (0.5 g), and urea (0.4 g) were dispersed in distilled water (20 mL) and stirred at 35 °C for 15 min. The pH of the mixture was then adjusted to 11. Subsequently, FXL (10 g) was incorporated into the mixture and thoroughly blended. The reaction proceeded at 120 °C for 2 h, after which the mixture was cooled to 25 °C, and the pH was adjusted to 6.5. The reaction product was precipitated and washed 2–3 times with 95 % ethanol. Finally, the precipitate was collected, dried, and ground to obtain PFXL.

### 2.3 Degree of substitution of phosphate groups determination (DS_P_) of PFXL and fourier transform infrared spectroscopy (FTIR) analysis

The phosphorus amount in PFXL was evaluated through the molybdenum blue colorimetry method (Zhao et al., 2022). A standard curve was generated with varying concentrations of potassium dihydrogen phosphate. PFXL (0.5 g) was incorporated into 7.5 mL of a sulfuric acid-nitric acid mixture (1:1 *v*/v) and digested until the solution became clear. After cooling to room temperature (25 °C), 2 mL of ammonium molybdate solution (50 g/L), 1 mL of sodium sulfite solution (200 g/L), and 1 mL of hydroquinone solution (5 g/L) were incorporated and uniformly blended. The solution was diluted to 25 mL and left to rest for 30 min, and the absorbance was then determined at 660 nm. The DSp of the phosphate group was computed using the formula (1):

(1) $${\mathrm{DS}}_{P}=162\times P/\left(3100-10\text{3P}\right)$$

P indicates the percent of phosphorus contents in PFXL.

The structural characteristics of PFXL were analyzed using FTIR (VERTEX70, Bruker Corporation, Karlsruher, Germany) as described by Meng et al. (2020). Each sample was scanned 64 times with a resolution of 4 cm^−1^ in a range from 4000 to 400 cm^−1^.

### 2.4 The effects of FXL and PFXL on the physicochemical properties of WS

#### 2.4.1 Pasting characteristics

The gelatinization characteristics of WS, WS-FXL, and WS-PFXL solutions were measured by a Brabender viscometer (Brabender GmbH & Co. KG, Germany). The solutions were prepared as follows: A specific quantity of WS was incorporated into 100 mL of distilled water, then FXL or PFXL was dispersed into the solution at concentrations of 0 %, 5 %, 10 %, 15 %, 20 %, and 25 % based on WS dry weight so that the total weight of solids in the 100 mL solution was 10 g. The solutions were heated from 50 °C to 95 °C at a rate of 1.5 °C/min, kept at 95 °C for 15 min, subsequently cooled from 95 °C to 50 °C at an equal rate, and maintained at 50 °C for 15 min. The pasting curves and parameters of the samples were documented through the Brabender viscometer software (Kong, Zhu, Zhang, & Zhu, 2020).

#### 2.4.2 Rheological properties

##### 2.4.2.1 Dynamic rheological measurement

The WS paste made in Section 2.4.1 was immediately transferred to a rheometer (TA DHR-2, Waters Co., USA) fitted with a 40 mm plate and a 1000 μm gap for viscoelasticity analysis. The storage modulus (G′) and loss modulus (G′′) were determined at 1 % strain over an oscillation range of 0.1–10 Hz (Xu et al., 2023).

##### 2.4.2.2 Static rheological measurement

The apparent viscosity and shear stress of the starch paste were measured across a shear rate range of 0.1–100 s^−1^ using the rheometer's static shear mode. The shear stress data were fitted through the Power-Law model (Zheng et al., 2024).

(2) $$\tau =K\times \gamma^{n}$$

The letters in the formula respectively represent shear stress (*τ*), consistency coefficient (*K*), shear rate (*γ*), and flow behavior index (*n*).

#### 2.4.3 Scanning electron microscopy (SEM)

The sample paste collected in Section 2.4.1 was saved at 4 °C for 24 h and dried using a freeze dryer. The freeze-dried gels were then sliced and coated with a 10 mm gold film on their cross-sections. The microstructure of starch gels was observed using SEM (Hitachi High-Tech Group, Japan) at an accelerating voltage of 15 kV and a magnification of 100× (Yang et al., 2022).

### 2.5 The effects of FXL and PFXL on the retrogradation behaviors of WS

#### 2.5.1 Hardness analysis

The sample paste produced in Section 2.4.1 was stored at 4 °C for various durations (1–14 days). Before measuring hardness, the gels were maintained at 25 °C for 30 min. Gel hardness was assessed utilizing a texture analyzer (TA.XT Express, SMS, UK) under the following conditions: a P/36R probe, pre-test, mid-test, and post-test speeds of 1.0 mm/s, a 40 % compression ratio, and a compression pause time of 5 s (Ren et al., 2020).

#### 2.5.2 Water distribution determination

Moisture distribution in the gels was analyzed by low-field nuclear magnetic resonance (LF-NMR, NMI20–015 V-I, Shanghai Niumag Electronic Technology, China). The experimental parameters were set as follows: T_W_ = 5000 ms, T_E_ = 0.8 ms, NECH = 15,000, and N_S_ = 2 (Li et al., 2019). The relaxation time spectrum (T_2_) was obtained and then inverted. The relative contents of tightly bound water (A_21_), semi-bound water (A_22_), and free water (A_23_) were computed based on the peak areas of the T_2_ inversion spectrum.

#### 2.5.3 X-ray diffraction (XRD)

The gels were freeze-dried after storage at 4 °C during varying durations (1–14 days) and subsequently ground into powder. Crystallinity analysis was performed by XRD (Model D8-Advance, Bruker AXS, Inc., Germany). The test parameters were set as follows: a diffraction range of 5° to 40°, a current of 40 mA, and a scanning rate of 4°/min. The relative crystallinity of the starch was measured by Jade 6.5 software (Fu et al., 2021).

#### 2.5.4 FTIR

FTIR (VERTEX70, Bruker Corporation, Karlsruhe, Germany) was employed to analyze the short-range order structures of the starch gel. Lyophilized gel powder was blended with potassium bromide (1:100), milled, and pressed for the FTIR test (Zhang et al., 2023). The test parameters were aligned with those detailed in Section 2.3.

### 2.6 Statistical analysis

All data were handled with SPSS 26 software, and means were compared by Duncan's multiple range test (*P* < 0.05). All figures, except for SEM images, were created through Origin 2022 software.

## 3 Results and discussion

### 3.1 DS_P_ and FT-IR spectra of PFXL

The phosphorus amount of PFXL was found to be 0.36 %, below the threshold of 0.4 %, which is regarded as safe in foods (Code of Federal Regulations, 2013). The DS_P_ of phosphate groups was computed to be 0.018, indicating a low level of phosphate group substitution.

**Fig. 1:**
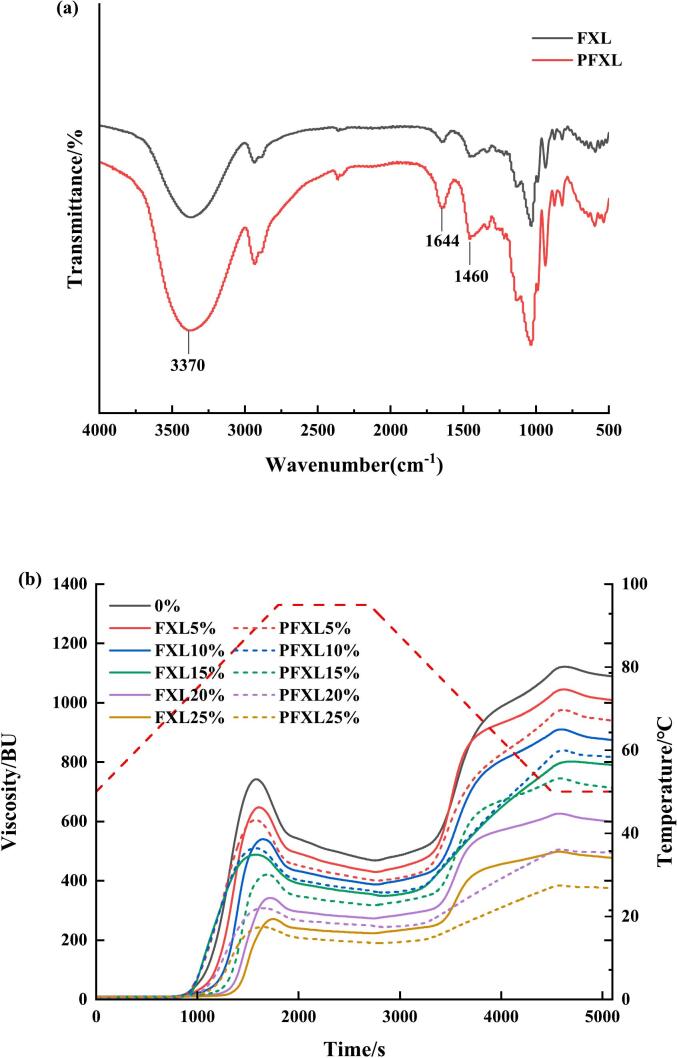
FTIR spectra of FXL and PFXL (a), Pasting curve of WS, WS-FXL, and WS-PFXL (b). Note: 0 % is WS without the addition of FXL and PFXL. FXL5 % is WS with 5 % FXL; PFXL5 % is WS with 5 % PFXL. FXL10 % is WS with 10 % FXL; PFXL10 % is WS with 10 % PFXL. FXL15 % is WS with 15 % FXL; PFXL15 % is WS with 15 % PFXL. FXL20 % is WS with 20 % FXL; PFXL20 % is WS with 20 % PFXL. FXL25 % is WS with 25 % FXL; PFXL25 % is WS with 25 % PFXL. The FXL/PFXL 5 %–25 % mentioned above is based on the dry weight of WS.

Fig. 1a. illustrates the FTIR spectrum of FXL and PFXL, which showed slight changes for PFXL compared to FXL. The intensity of the absorption peak at 3370 cm^−1^ increased, indicating O—H stretching vibrations enhanced. This suggested that more –OH groups were likely introduced into the PFXL molecule, therefore improving its water solubility. The absorption peak at 1644 cm^−1^ corresponded to the asymmetric stretching vibration of C=O, which indicated the existence of carboxyl groups in the FXL structure. The peak at 1460 cm^−1^ was associated with C—H absorption, which was the characteristic peak of polysaccharides (Tang et al., 2024). Notably, PFXL did not alter the original peak structure of FXL, and the characteristic vibrational bands of phosphorylated polysaccharides (P–O–C and P=O) were not observed, which was aligned with the findings of Yang et al. (2016). It might result from the low degree of phosphate substitution or the strong vibrations of other absorption peaks in PFXL, which resulted in signal overlap and obscured the characteristic vibration bands (Gao et al., 2014; Wu et al., 2022).

### 3.2 Pasting properties

Pasting properties reflect the viscosity, thermal stability, and short-term aging of starch granules. In the process of pasting, starch granules absorb water, swell, and amylose leach (Rong et al., 2022). Fig. 1b and Table 1 indicated the gelatinization curves and parameters of WS added with PFXL and FXL, respectively. Table 1 indicated that incorporation of PFXL and FXL remarkably influenced the gelatinization parameters of WS, resulting in marked reductions in peak viscosity (PV), trough viscosity (TV), final viscosity (FV), breakdown value (BD), and setback value (SB). These reductions suggested a positive correlation with the concentration of PFXL and FXL. The observed decrease in WS viscosity was consistent with the findings of Huang, Yu, Wang, & Shi, 2023, who considered that jicama polysaccharide prevented the pasting of starch granules by contending with starch for water, thereby reducing its viscosity. The decline in PV, TV, and FV can be explained by the lower viscosity of PFXL and FXL due to their lower molecular weight compared to WS (Ji et al., 2020). Besides, the PV value of WS-PFXL was lower compared to WS-FXL at an equivalent addition level. This might be attributed to the stronger hydrophilicity of PFXL, which could enhance its competition with WS for water during pasting, leading to a further reduction in WS viscosity.

**Table 1:** Pasting parameters of WS, WS-FXL and WS-PFXL.

Sample	PV(BU)	TV(BU)	FV(BU)	BD(BU)	SB(BU)
0%	739.0 ± 4.2^a^	470.0 ± 1.4^a^	1107.0 ± 7.1^a^	269.0 ± 2.8^a^	637.0 ± 8.5^a^
FXL5%	630.0 ± 25.5^b^	421.5 ± 16.3^b^	1000.0 ± 38.2^b^	208.5 ± 9.2^b^	578.5 ± 21.9^b^
FXL10%	535.5 ± 0.7^d^	373.0 ± 5.7^d^	901.0 ± 4.2^d^	162.5 ± 4.9^c^	502.0 ± 8.5^d^
FXL15%	424.0 ± 4.2^e^	315.5 ± 3.5^e^	734.0 ± 4.2^f^	108.5 ± 7.8^d^	418.5 ± 0.7^f^
FXL20%	345.0 ± 4.2^f^	277.0 ± 4.2^f^	623.5 ± 3.5^g^	68.0 ± 0.0^e^	346.5 ± 0.7^g^
FXL25%	271.5 ± 0.7^h^	224.0 ± 1.4^h^	500.5 ± 9.2^h^	47.5 ± 0.7^f^	276.5 ± 7.8^h^
PFXL5%	601.5 ± 3.5^c^	403.5 ± 0.7^c^	957.0 ± 1.4^c^	198.0 ± 2.8^b^	553.5 ± 0.7^c^
PFXL10%	516.0 ± 8.5^d^	363.0 ± 5.7^d^	811.0 ± 13.4^e^	153.0 ± 14.1^c^	448.5 ± 7.8^e^
PFXL15%	407.5 ± 0.7^e^	309.5 ± 2.1^e^	652.0 ± 4.2^g^	98.0 ± 1.4^d^	342.5 ± 2.1^g^
PFXL20%	316.0 ± 11.3^g^	251.0 ± 2.8^g^	500.5 ± 4.9^h^	65.0 ± 8.5^e^	249.5 ± 2.1^i^
PFXL25%	246.0 ± 2.8^i^	192.5 ± 2.1^i^	372.5 ± 7.8^i^	53.5 ± 0.7^ef^	180.0 ± 9.9^j^

Values are presented as means ± SD. Means in each column with different superscript letters indicate significant differences. (P < 0.05).

PV: Peak viscosity; TV: Trough viscosity; FV: Final viscosity; BD: Breakdown; SB: Setback.

Note: 0% is WS without the addition of FXL and PFXL.

FXL5 % is WS with 5 % FXL; PFXL5% is WS with 5% PFXL.

FXL10 % is WS with 10 % FXL; PFXL10 % is WS with 10% PFXL.

FXL15 % is WS with 15 % FXL; PFXL15% is WS with 15% PFXL.

FXL20 % is WS with 20 % FXL; PFXL20% is WS with 20% PFXL.

FXL25 % is WS with 25 % FXL; PFXL25 % is WS with 25 % PFXL.

The FXL/PFXL 5%–25% mentioned above is based on the dry weight of WS.

The BD value, determined by subtracting TV from PV, serves as a critical indicator for assessing starch granule stability under thermal and shear stress conditions (Zhang et al., 2019). Table 1 displayed that PFXL and FXL significantly lowered the BD value of WS. However, no notable statistical difference in BD was detected between PFXL and FXL under the same conditions. This suggested that FXL improved the thermal instability of WS, irrespective of phosphorylation modification.

The SB signifies the reaggregation of pasting starch molecules (particularly amylose) in the cooling phase, which reflects the short-term aging of WS (Luo, Shen, et al., 2020). It was observed that both PFXL and FXL significantly minimized the SB value of WS, suggesting that they played a positive effect in delaying WS short-term aging. This finding aligned with our previous research, where FI, FS, and FXL all markedly delayed the short-term retrogradation of WS when addition levels were less than 15 % (Luo et al., 2017). Notably, PFXL exhibited a stronger inhibitory effect than FXL, and this effect became more prominent with higher addition. When the amount added ranged from 5 % to 25 %, the SB value of FXL decreased by 52.2 %, whereas it decreased by 67.5 % for PFXL. The short-term aging of WS is primarily due to amylose recrystallization. The phosphorylation modification improved the water-binding and water-retention capacity of FXL, enabling it to more effectively compete with amylose for available water and partially disrupting the amylose-amylose interactions. This declined the degree of amylose gelation, thereby providing stronger inhibition in the short-term aging of WS (Zhou et al., 2022).

### 3.3 Rheological properties

#### 3.3.1 Dynamic rheological properties

**Fig. 2:**
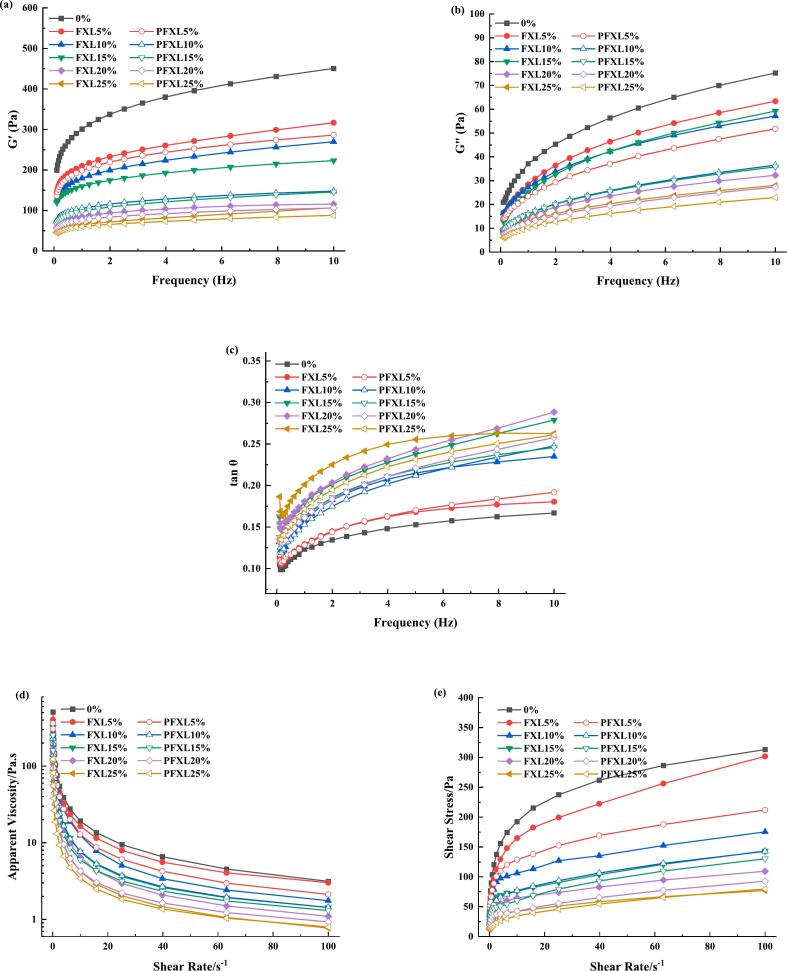
Effect of FXL and PFXL on the rheological properties of WS paste. (a)-Storage modulus, G′; (b)-loss modulus, G′′; (c)-loss tangent, tan θ; (d)-apparent viscosity; (e)-stress. Note: 0 % is WS without the addition of FXL and PFXL. FXL5 % is WS with 5 % FXL; PFXL5 % is WS with 5 % PFXL. FXL10 % is WS with 10 % FXL; PFXL10 % is WS with 10 % PFXL. FXL15 % is WS with 15 % FXL; PFXL15 % is WS with 15 % PFXL. FXL20 % is WS with 20 % FXL; PFXL20 % is WS with 20 % PFXL. FXL25 % is WS with 25 % FXL; PFXL25 % is WS with 25 % PFXL. The FXL/PFXL 5 %–25 % mentioned above is based on the dry weight of WS.

Fig. 2a and b illustrated the influence of PFXL and FXL on the dynamic frequency scanning of WS paste. The parameters G′ and G′′ represent the elastic and the viscous modulus, respectively, while the tan θ represents the ratio of viscosity to elasticity. Overall, both G′ and G′′ values enhanced with vibration frequency, and G′ consistently exceeded G′′ within 0.1–10 Hz range. With PFXL and FXL incorporated, G′ and G′′ values of WS paste decreased, indicating a significant reduction in its viscoelastic properties. Since amylose is the primary polymer generating the cross-linking network (Yuris et al., 2017), PFXL and FXL may interact with amylose, weakening amylose-amylose interactions, thereby delaying amylose reaggregation and reducing the G′ and G′′ values of WS paste (Kong, Zhu, Zhang, & Zhu, 2020). Notably, PFXL exhibited lower G′ and G′′ values compared to FXL, suggesting a stronger interaction of PFXL with amylose, which more effectively disrupted the interactions among amylose and starch chains. Additionally, tan θ remained below 1 for samples (Fig. 2c) and rose with the incorporation of PFXL and FXL, indicating a more pronounced decrease in G′ compared to G′′. This observation was consistent with findings on the effects of bamboo shoot polysaccharides on lotus root starch (Zheng et al., 2024).

#### 3.3.2 Static rheological measurement

Fig. 2d and e showed the apparent viscosity and shear stress curves for WS, WS-FXL, and WS-PFXL, respectively. The viscosity of the three systems diminished as the shear rate demonstrated typical shear-thinning behavior (Liu et al., 2023). As the levels of PFXL and FXL increased, the viscosity gradually declined. This could be attributed to the ability of PFXL and FXL to create steric hindrance around the starch, preventing WS from absorbing water and limiting the starch particle expansion. Furthermore, at equivalent addition levels, PFXL exhibited a greater impact in reducing the apparent viscosity of WS than FXL. This was likely due to the higher hydrophilicity of PFXL, which enhanced its ability to compete for water, thereby restricting starch swelling. Table 2 presents the shear stress curve results for the three systems fitted by the Power Law model. The flow behavior index (n) was below 1 for all samples, indicating pseudoplastic behavior (Kong, Zhu, Zhang, & Zhu, 2020). Both the consistency coefficient (K) and shear stress progressively decreased with increasing levels of FXL and PFXL, further confirming that the incorporation of PFXL and FXL decreased the viscosity and strength of the WS paste.

**Table 2:** Rheological parameters of WS, WS-FXL and WS-PFXL mixtures.

Sample	K(Pa·s^n^)	n (−)	R^2^
0%	105.050 ± 0.156^a^	0.245 ± 0.000^bcd^	0.991
FXL5%	71.915 ± 16.464^b^	0.283 ± 0.010^ab^	0.995
FXL10 %	64.881 ± 0.977^b^	0.223 ± 0.011^cd^	0.995
FXL15%	42.495 ± 11.271^cd^	0.244 ± 0.050^bcd^	0.973
FXL20%	40.955 ± 4.516^cd^	0.212 ± 0.018^d^	0.985
FXL25%	29.302 ± 1.949^def^	0.206 ± 0.001^d^	0.982
PFXL5%	74.373 ± 2.460^b^	0.226 ± 0.006^cd^	0.992
PFXL10%	48.558 ± 2.695^c^	0.221 ± 0.016^cd^	0.991
PFXL15%	36.195 ± 2.081^cde^	0.264 ± 0.015^bc^	0.979
PFXL20%	25.533 ± 1.604^ef^	0.261 ± 0.009^bc^	0.985
PFXL25%	17.410 ± 0.513^f^	0.316 ± 0.004^a^	0.989

K: consistency coefficient; n: flow behavior index.

Note: 0% is WS without the addition of FXL and PFXL.

FXL5% is WS with 5% FXL; PFXL5% is WS with 5% PFXL.

FXL10% is WS with 10 % FXL; PFXL10% is WS with 10% PFXL.

FXL15% is WS with 15% FXL; PFXL15% is WS with 15% PFXL.

FXL20% is WS with 20% FXL; PFXL20% is WS with 20% PFXL.

FXL25% is WS with 25% FXL; PFXL25% is WS with 25% PFXL.

The FXL/PFXL 5%–25% mentioned above is based on the dry weight of WS.

### 3.4 SEM

**Fig. 3:**
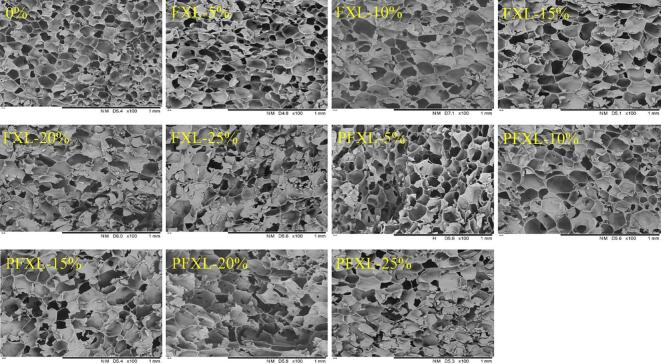
Effects of FXL and PFXL on the surface morphology of WS paste after freeze drying. Note: 0 % is WS without the addition of FXL and PFXL. FXL5 % is WS with 5 % FXL; PFXL5 % is WS with 5 % PFXL. FXL10 % is WS with 10 % FXL; PFXL10 % is WS with 10 % PFXL. FXL15 % is WS with 15 % FXL; PFXL15 % is WS with 15 % PFXL. FXL20 % is WS with 20 % FXL; PFXL20 % is WS with 20 % PFXL. FXL25 % is WS with 25 % FXL; PFXL25 % is WS with 25 % PFXL. The FXL/PFXL 5 %–25 % mentioned above is based on the dry weight of WS.

The microstructures of WS, WS-FXL, and WS-PFXL gels freeze-dried after short-term storage (1 d) were displayed in Fig. 3. Each gel exhibited a characteristic three-dimensional honeycomb structure. During cooling, WS paste formed a network that encapsulates water, which was subsequently lost during freeze-drying. The WS gel displayed a dense, intact network with smaller pores, which varied depending on the addition levels of PFXL and FXL. At lower addition levels (≤5 %), PFXL and FXL had minimal impact on the WS gel structure. However, as the addition increased, the gel structure became noticeably less intact and orderly. Some deep honeycombs were covered by small fragments, a phenomenon that became more prominent at higher addition levels. These small fragments were likely formed by PFXL or FXL, which combined with WS during the pasting and adhered to or participated in the network formation during cooling (Zheng et al., 2024). PFXL, in particular, induced more extensive coverage of the deep honeycomb structure than FXL, which helps reduce water loss during storage and delayed starch recrystallization. This suggested that PFXL was more effective than FXL in inhibiting WS retrogradation, consistent with the findings of Section 3.2. Simultaneously, this implied a stronger interaction between PFXL and WS, which resulted from the properties of FXL modified by phosphorylation. PFXL had a larger molecular weight and more hydrophilic hydroxyl groups, which were prone to interact with starch.

### 3.5 Gel hardness

**Fig. 4:**
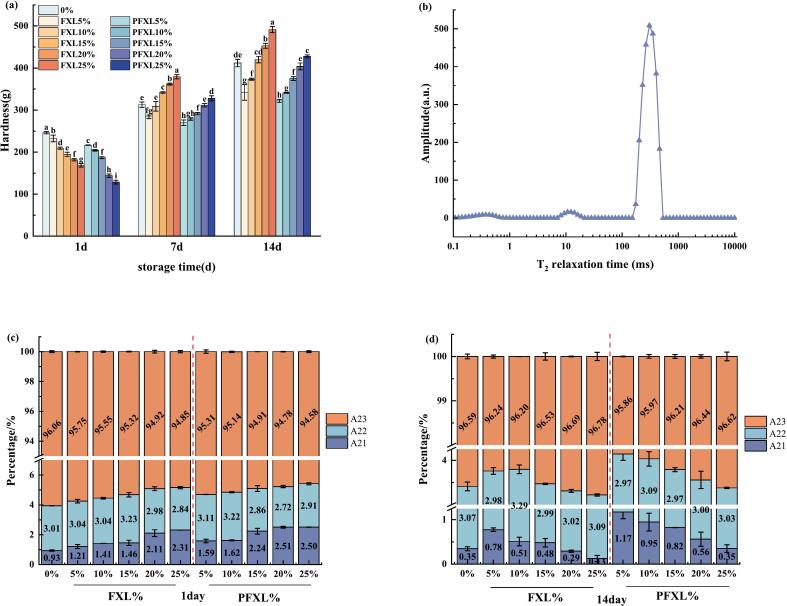
Effects of FXL and PFXL on the hardness of WS gel during storage time (a), and the peak area ratio of different water during WS aging (b-d). Note: 0 % is WS without the addition of FXL and PFXL. FXL5 % is WS with 5 % FXL; PFXL5 % is WS with 5 % PFXL. FXL10 % is WS with 10 % FXL; PFXL10 % is WS with 10 % PFXL. FXL15 % is WS with 15 % FXL; PFXL15 % is WS with 15 % PFXL. FXL20 % is WS with 20 % FXL; PFXL20 % is WS with 20 % PFXL. FXL25 % is WS with 25 % FXL; PFXL25 % is WS with 25 % PFXL. The FXL/PFXL 5 %–25 % mentioned above is based on the dry weight of WS.

The hardness of WS, WS-FXL, and WS-PFXL gels preserved at 4 °C for 1, 7, and 14 days was measured using a texture analyzer (Fig. 4a). The gel hardness gradually increased over the storage period due to water loss (Zheng et al., 2024). Both PFXL and FXL had a distinct influence on the hardness of WS gels, depending on storage time and addition levels. After short-term storage (1 day), adding PFXL and FXL significantly diminished the hardness of WS gels, with PFXL demonstrating a more pronounced effect than FXL. This indicated that PFXL was more potent for delaying the short-term retrogradation of WS gels, following the findings in Section 3.2. The introduction of 25 % PFXL maximally reduced the viscosity of WS paste, inhibited amylose retrogradation, and consequently lowered the gel hardness. When stored for a long period (7 d), PFXL and FXL only decreased WS gel hardness at lower addition levels. However, when the addition level of PFXL and FXL, respectively, exceeded 10 % and 20 %, an opposite trend was observed. This influence was thought to be the water precipitation from WS gels due to the aging, which was more pronounced at a long storage time (14 d) and higher addition levels (20 %–25 %) (Zhou et al., 2022). Overall, PFXL exhibited a stronger capacity than FXL in preventing the increase of gel hardness, regardless of storage time or addition level. This can be ascribed to PFXL's superior water retention ability, which minimized water loss in the gel during storage and inhibited WS retrogradation through hydrogen bonding interactions. (Liu et al., 2023).

### 3.6 LF-NMR analysis

LF-NMR is considered a quick and efficient approach for measuring water distribution during starch aging. Fig. 4b displayed the relaxation time (T_2_) profile of WS gel, where the three peaks correspond to different water types in the gel. The relaxation time falling within 0.1–10 ms represents tightly bound water (T_21_), 10–100 ms corresponds to semi-bound water (T_22_), and greater than 100 ms indicates free water (T_23_) with the highest molecular mobility and percentage (Li et al., 2019).

The relative amounts of the three kinds of water in WS, WS-FXL, and WS-PFXL gels were derived by inverting the T_2_ spectrum, with results shown in Fig. 4c and d. After short storage (1 day), the portion of tightly bound water (A_21_) grew, while free water (A_23_) decreased in the WS gel system with increasing amounts of PFXL and FXL, in agreement with the results of Ma et al. (2019). PFXL and FXL could bind more water molecules and restrict water migration due to their hydrophilicity, thereby reducing the proportion of A_23_ (Zhou et al., 2022). At equivalent addition levels, A_23_ in PFXL gels was consistently lower than in FXL gels, likely because of the incorporation of phosphate groups in PFXL, which enhanced hydrophilicity and water retention capacity. The percentage of semi-bound water (A_22_) initially elevated and then dropped with rising PFXL and FXL additions. At low addition levels (≤10 % and ≤ 15 %), they could not form a gel by themselves, but their presence promoted water binding in WS, leading to the transfer of A_23_ to A_22_ and A_21_. In contrast, at higher addition levels (>10 % and > 15 %), they began to form weak gels on their own; therefore, their additions promoted the transfer of A_23_ and A_22_ to A_21_. Following extended storage (14 d), an inverse tendency was detected, with A_21_ decreasing and A_23_ increasing as FXL and PFXL levels rose, while A_22_ remained relatively stable. This shift was attributed to the significant retrogradation of WS during extended storage, leading to water precipitation from the gels and a higher proportion of A_23_. Notably, the A_23_ content in PFXL was significantly lower than in WS and FXL gels (except at 25 % addition), suggesting that PFXL more effectively inhibited WS retrogradation. Overall, across both short- and long-term storage, PFXL demonstrated superior suppression of WS gel retrogradation compared to FXL.

### 3.7 XRD

**Fig. 5:**
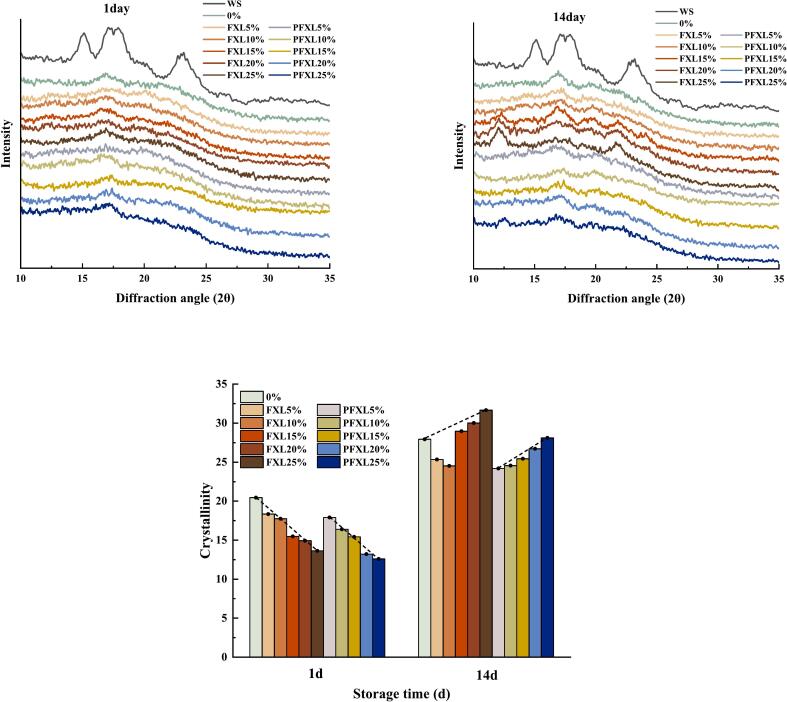
Effects of FXL and PFXL on the crystalline of WS during different storage times. Note: WS is ungelatinized native starch granules. 0 % is WS without the addition of FXL and PFXL. FXL5 % is WS with 5 % FXL; PFXL5 % is WS with 5 % PFXL. FXL10 % is WS with 10 % FXL; PFXL10 % is WS with 10 % PFXL. FXL15 % is WS with 15 % FXL; PFXL15 % is WS with 15 % PFXL. FXL20 % is WS with 20 % FXL; PFXL20 % is WS with 20 % PFXL. FXL25 % is WS with 25 % FXL; PFXL25 % is WS with 25 % PFXL. The FXL/PFXL 5 %–25 % mentioned above is based on the dry weight of WS.

XRD is utilized to evaluate the long-range orderliness for WS. Native WS exhibited prominent peaks at 17.1°, 18°, and 23.0°, characteristic of an A-type crystallization pattern. Fig. 5 illustrates the effects of FXL and PFXL additions on the crystallinity of WS stored at 4 °C for different storage periods (1 day and 14 days). The findings displayed that no new diffraction peaks emerged with the introduction of FXL and PFXL, indicating that the crystallization type of the starch remained unchanged.

The crystallinity of WS, WS-FXL, and WS-PFXL significantly increased as the preservation period prolonged from 1 day to 14 days, owing to WS aging. After short-term storage (1 day), the introduction of PFXL and FXL decreased the crystallinity of WS, aligned with the results of Luo, Xiao, et al. (2020). The reduction was more pronounced at higher addition levels, with PFXL exhibiting a greater effect than FXL. However, after extended storage (14 days), a noticeable shift in crystallinity reduction was observed. In contrast, the reduction of the crystallinity decreased with the increase of PFXL and FXL. PFXL consistently reduced the crystallinity of starch within the studied addition range (5 %–25 %), while FXL only had this effect at lower levels (5 %–10 %). Overall, PFXL demonstrated more effective suppression of WS retrogradation than FXL, consistent with the pasting property results.

In Fig. 5, the peaks at 13° and 20° were the signature of amylopectin, while the peak at 17° corresponded to amylose (Ji et al., 2022). The diffraction peaks at 13° and 20° were not observed after the addition of PFXL, indicating that PFXL inhibited the amylopectin recrystallization. Conversely, when the FXL concentration exceeded 15 %, diffraction peaks of 13° and 20° were observed in WS, and their intensity increased with rising FXL levels, suggesting that excessive FXL promoted amylopectin recrystallization (Luo et al., 2017). This distinction was attributable to the evident physicochemical properties of PFXL and FXL. The water retention capacity of FXL was improved by the phosphorylation modification, which could impede water precipitation from WS-PFXL gel during long-term storage. Phosphorylation also reduced the relative crystallinity of PFXL, particularly at higher concentrations (Liu et al., 2022; Wu et al., 2022). In addition, PFXL contained more hydroxyl groups, allowing it to form hydrogen bonds with starch. These factors collectively contribute to the reduced ability of amylopectin to recrystallize.

### 3.8 FTIR

**Fig. 6:**
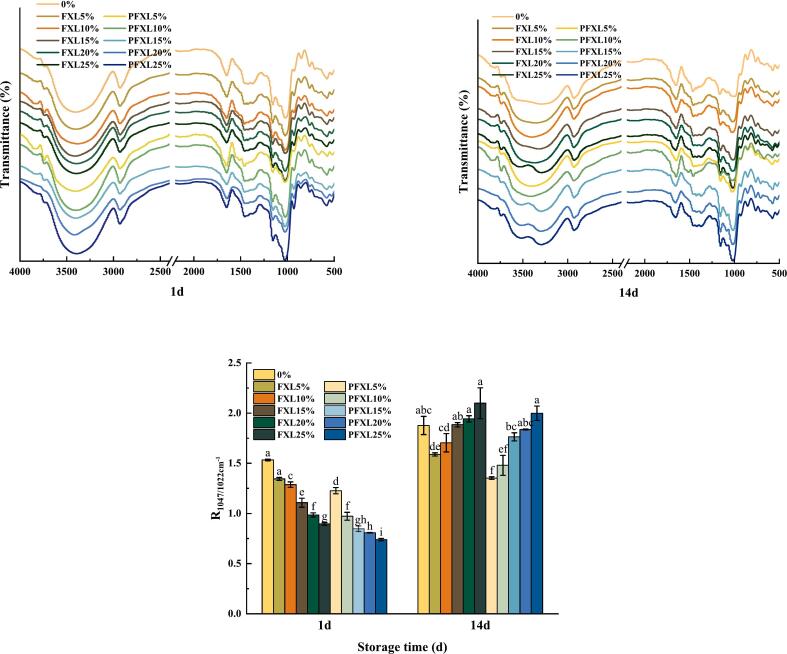
FTIR spectra of WS, WS-FXL and WS-PFXL gels dried by freeze drying. Note: 0 % is WS without the addition of FXL and PFXL. FXL5 % is WS with 5 % FXL; PFXL5 % is WS with 5 % PFXL. FXL10 % is WS with 10 % FXL; PFXL10 % is WS with 10 % PFXL. FXL15 % is WS with 15 % FXL; PFXL15 % is WS with 15 % PFXL. FXL20 % is WS with 20 % FXL; PFXL20 % is WS with 20 % PFXL. FXL25 % is WS with 25 % FXL; PFXL25 % is WS with 25 % PFXL. The FXL/PFXL 5 %–25 % mentioned above is based on the dry weight of WS.

FTIR was employed to further investigate the short-range orderliness of the WS gels (Fig. 6). The gels exhibited an absorption peak in the 3396–3426 cm^−1^ range, ascribed to O—H stretching vibrations, representing interactions between WS and water (Wei et al., 2023). The peaks at 1019–1020 and 1080–1081 cm^−1^ corresponded to bending vibrations of C—H and C–O–H (Zhou et al., 2022). Notably, no additional absorption peaks appeared in WS-PFXL relative to WS. The peaks between 800 and 1200 cm^−1^ were related to the stretching and vibrations of C—O, C—C, and C–O–H, which characterize the crystalline structure of starch. Absorption peaks at 1047 cm^−1^ and 1022 cm^−1^ indicate the crystalline and amorphous regions of starch, individually. The absorbance ratio at 1047/1022 cm^−1^ is commonly applied to assess the degree of short-range order in starch. The effects of FXL and PFXL on the order degree of starch aligned with the XRD results, further confirming that PFXL was more efficient than FXL in inhibiting the long-term retrogradation of WS.

## 4 Conclusion

Phosphorylation-modified FXL demonstrated a marked influence on the physicochemical characteristics and retrogradation behavior of WS compared to unmodified FXL. PFXL significantly reduced the peak viscosity, trough viscosity, final viscosity, breakdown value, setback value, G′, and G′′, and apparent viscosity of WS. At higher addition levels (>5 %), PFXL formed small fragments that covered the three-dimensional honeycomb structure of WS. Compared to FXL, PFXL had a greater impact in reducing WS gel hardness during both short and long storage. Additionally, PFXL consistently decreased the crystallinity of WS during a long storage time (14 days) within the addition range of 5 %–25 %, whereas FXL had this effect only at lower additions (5 %–10 %). In conclusion, FXL modified by phosphorylation exhibited significant advantages in improving the properties of WS, particularly in inhibiting its aging. This study enhanced the application of FXL in WS and provided a reference for the use of PFXL in WS and WS-based foods.

## CRediT authorship contribution statement

**Ruijie Zhang:** Writing – original draft. **Denglin Luo:** Methodology, Data curation. **Chonghui Yue:** Investigation. **Zhouya Bai:** Methodology, Conceptualization. **Peiyan Li:** Validation. **Libo Wang:** Supervision, Resources. **Sihai Han:** Writing – review & editing.

## Declaration of competing interest

The authors declare that they have no known competing financial interests or personal relationships that could have appeared to influence the work reported in this paper.

## Data Availability

Data will be made available on request.
